# USP36-Mediated Deubiquitination of DOCK4 Contributes to the Diabetic Renal Tubular Epithelial Cell Injury *via* Wnt/β-Catenin Signaling Pathway

**DOI:** 10.3389/fcell.2021.638477

**Published:** 2021-04-23

**Authors:** Suwei Zhu, Shaoshuai Hou, Yao Lu, Wei Sheng, Zhengguo Cui, Tianyi Dong, Hong Feng, Qiang Wan

**Affiliations:** ^1^Department of Nephrology, Shandong Provincial Hospital, Cheeloo College of Medicine, Shandong University, Jinan, China; ^2^Department of Endocrinology, Shandong Provincial Hospital Affiliated to Shandong First Medical University, Jinan, China; ^3^Department of Cancer Center, Shandong Provincial Hospital, Cheeloo College of Medicine, Shandong University, Jinan, China; ^4^Department of Public Health, Graduate School of Medicine and Pharmaceutical Sciences, University of Toyama, Toyama, Japan; ^5^Department of Breast and Thyroid Surgery, Shandong Provincial Hospital Affiliated to Shandong First Medical University, Jinan, China; ^6^Department of Cancer Center, Shandong Provincial Hospital Affiliated to Shandong First Medical University, Jinan, China; ^7^Department of Endocrinology, Shandong Provincial Hospital, Cheeloo College of Medicine, Shandong University, Jinan, China

**Keywords:** ubiquitin specific proteases 36, dedicator of cytokinesis 4, Wnt/β-catenin, diabetic kidney disease, deubiquitination, epithelial-to-mesenchymal transition

## Abstract

Diabetic kidney disease (DKD) has become the leading cause of end-stage renal disease but the efficacy of current treatment remains unsatisfactory. The pathogenesis of DKD needs a more in-depth research. Ubiquitin specific proteases 36 (USP36), a member of deubiquitinating enzymes family, has aroused wide concerns for its role in deubiquitinating and stabilizing target proteins. Nevertheless, the role of USP36 in diabetes has never been reported yet. Herein, we identified an increased expression of USP36 both *in vitro* and *in vivo* in diabetic renal tubular epithelial cells (TECs), and its overexpression is related to the enhanced epithelial-to-mesenchymal transition (EMT). Further investigation into the mechanisms proved that USP36 could directly bind to and mediate the deubiquitination of dedicator of cytokinesis 4 (DOCK4), a guanine nucleotide exchange factor (GEF) that could activate Wnt/β-catenin signaling pathway and induce EMT. Our study revealed a new mechanism that USP36 participates in the pathogenesis of DKD, and provided potential intervening targets accordingly.

## Introduction

Along with the worldwide prevalence of diabetes, the incidence of its major microvascular complication diabetic kidney disease (DKD) increased rapidly, and currently DKD has become the leading cause of end-stage renal disease (ESRD) (Li et al., 2017). Despite the extensive investigations and progresses were achieved in the pathogenic mechanisms of DKD during the past decades, no curative agents for DKD are clinically available yet. Current treatments only focus on the risk factors mediating progression of DKD, such as hyperglycemia, hypertension and proteinuria (Nørgaard et al., 1990; de Boer et al., 2011; Mise et al., 2015). A further exploration of the pathogenesis of DKD, and accordingly potential intervening targets, is still of significance.

Ubiquitin–proteasome system (UPS) is one of the major protein degrading mechanisms for eukaryotic cells, mainly responsible for mediating the degradation of cellular short-lived proteins, damaged or misfolded proteins (Reyes-Turcu et al., 2009). Along with the recognition of the roles USP playing in modulating cell cycle progression, gene expression, cell survival and apoptosis, UPS emerged as one crucial modulator for cellular morphological and functional homeostasis (Meyer-Schwesinger, 2019). Within kidneys, UPS was also evidenced to participate in regulating ample kidney biological functions, including erythropoiesis (Meyer-Schwesinger, 2019), glucose reabsorption (Hartleben et al., 2013), salt and water balance (Wu et al., 2018). Besides, accumulating data suggested that UPS serves as an active participant in the pathogenesis of several lines of kidney pathologies, such as glomerulonephritis (Bontscho et al., 2011), acute kidney injuries (Palombella et al., 1994) and renal fibrosis (Fukasawa, 2012). However, whether UPS is also involved in the pathogenesis of DKD gained little attention, and limited studies concerning this issue reached inconsistent conclusions yet. For example, a study reported that UPS was enhanced in DKD and high glucose stimulation might promote proteasomal activities (Aghdam et al., 2013), while another report suggested that proteasomal activities were reduced in DKD (Portero-Otín et al., 1999). The concrete role of UPS in DKD still remains exploration.

Recently, ubiquitin specific proteases 36 (USP36), a member which belongs to deubiquitinating enzymes family, has aroused wide concerns for its well-identified effects in stabilizing and modifying c-Myc (Sun et al.,2015a,b). Subsequent studies revealed that USP36 is also capable of reducing Histone H2B ubiquitination at p21 locus, thereby enhancing p21 signaling and regulating cell proliferation (DeVine et al., 2018). Besides, USP36 is known to locate within mitochondria and modulate mitochondrial respiration *via* deubiquitinating superoxide dismutase 2 (SOD2) (Kim et al., 2011). Accompanied with the identifications of these biological functions of USP36, its role in tumorigenesis such as ovarian cancer (Li et al., 2008) and neuroblastoma (Mondal et al., 2018) has been established as well. Our previous study reported that USP36 is ubiquitously expressed within kidneys and its altered expressions participate in the pathogenesis of ischemic kidney injuries (Liu et al., 2019). Herein, we provided evidences of the increased expression of USP36 both *in vitro* in high glucose-induced renal tubular epithelial cells (TECs) and *in vivo* in human and murine DKD models, and its overexpression is related to the enhanced epithelial-to-mesenchymal transition (EMT). Further, we found that USP36 could directly bind to and mediate the deubiquitination of dedicator of cytokinesis 4 (DOCK4), a guanine nucleotide exchange factor (GEF) that could activate Wnt/β-catenin signaling pathway and induce EMT. Our study revealed a new mechanism that USP36 participates in the pathogenesis of DKD, and provided potential intervening targets accordingly.

## Materials and Methods

### Cell Culture and Treatment

Human renal proximal tubular epithelial cells (HK-2) were obtained from the American Type Culture Collection (ATCC, Manassas, VA) and cultured in DMEM medium (Gibco, United States) supplemented with 10% fetal bovine serum (Gibco, United States) and penicillin (100 U/ml) and streptomycin (100 mg/ml, Gibco, United States) in a humidified atmosphere of 5% CO2 at 37°C. Cells were cultured in medium containing either 5 mM (normal glucose, NG) or 25.5 mM (high glucose, HG) glucose for 72 h.

### Human Kidney Specimens

Renal histological sections were gained from the patients who were diagnosed as diabetic nephropathy. Human renal biopsy was done in the department of Pathology, Shandong University School of Medicine.

### Mouse Models

The male C57BL/6 mice (8 w, 21 ∼ 25 g) were purchased from the Shandong University Laboratory Animal Center, which were housed in controlled environments (temperature: 23 ± 2°C and exposure to light from 07:00–19:00) and supplied with a standard mouse chow and water *ad libitum*. All animal experiments were performed according to the protocols approved by Animal Ethics Committee of Shandong University. The mice were randomly divided into the following experimental groups: normal control mice, streptozotocin-induced DKD mice. DKD groups were given an intraperitoneal injection of streptozotocin (50 mg/kg) after 12 h fasting for five consecutive days. Control group treated with an equal volume of citric acid buffer solution. One week after STZ injection, mice with random blood glucose levels over 16.7 mM were considered diabetes. Four months after the diagnosis of diabetes, the mice were euthanized under anesthesia, and the kidneys were quickly removed and stored in Paraformaldehyde fixative fluid (Yu et al., 2016).

### SiRNAs and Plasmids

USP36 and DOCK4 siRNAs were constructed by RiboBio (Guangzhou, CN). USP36 siRNA5′-GGGACCAGCAACTCGAATA-3′, DOCK4 siRNA5′-CCAGCAACGTCTTGAACCA-3′. USP36 overexpression and control plasmids were purchased from Public Protein/Plasmid Library (PPL). Ribo FECT^TM^ CP Transfection KIT(C10511-05, RiboBio) and Lipofectamine 3000 (L3000-015, Invitrogen) were used as manufacturer’s instructions described.

### Western Blotting

After treatment, cells were washed with cool PBS for three times and lysed with RIPA buffer, containing protease and phosphatase inhibitor cocktail (P1260, Solarbio). The samples were incubated on ice for 30 min and then centrifuged for 30 min at 4°C at 12,000 rpm/min. Collected the protein supernatant. We used 8% SDS-PAGE to divide protein in different molecular weight then the protein was transferred into 0.45 μm PVDF membranes (Millipore, United States) by electroblot for 2 h. Membranes were blocked in TBST, containing 5% no-fat milk for 1 h and then incubated in primary antibodies at 4°C overnight. Primary antibodies were used as follows: rabbit anti-USP36 antibody (14783-1-AP, Proteintech,1:1,000), rabbit anti-DOCK4 antibody (21861-1-AP, Proteintech,1:1,000), rabbit anti-β-catenin antibody (51067-1-AP, Proteintech, 1:5,000), rabbit anti-E-cad antibody (20874-1-AP, Proteintech,1:5,000), rabbit anti-α-SMA antibody (55135-1-AP, Proteintech,1:2,000). And then the membranes were incubated with the corresponding second antibody (goat anti-rabbit IgG) and detected by enhanced chemiluminescence reagents (ECL, Millipore, United States) and analyzed by Image J software.

### Real-Time Polymerase Chain Reaction

Total RNA was extracted using TRIzol Reagent (Invitrogen, United States) as manufacturer’s instructions described and first strand cDNA synthesis was performed using Revert Aid First Strand cDNA Synthesis Kit (Thermo scientific). Real-time polymerase chain reaction (RT-qPCR) was carried out using Ultra SYBR Mixture kit (Cwbio, CW2601M) on Roche 480II as following: firstly, 95°C for 2 min to denaturation, the second 40 cycles at 95°C for 15 s, 60°C for 1 min, the third 95°C to melt and lastly 50°C for 30 s to cool down. 5′-AGCACTTTTCCCCCAGAACTG-3′ (forward) and 5′-GGCTCCCAGATCTGCTGCTA-3′ (reverse) for USP36. 5′-GAAGTGTGACGTGGACATCC-3′ (forward) and 5′-CCGATCCACACGGAGTACTT-3′ (reverse) for β-actin. 5′-GGGCAATGAACAACTGGGAC-3′ (forward) and 5′CCTCTCCCAGGTTGGAACAC-3′ (reverse) for DOCK4.

### Immunohistochemistry

The sections were baked in a 68°C oven for 2 h, and then dewaxed by gradient ethanol. For antigen retrieval, tissue sections were placed in sodium citrate buffer and boiled for 3 min. ORIGENE PV-9000 kit was performed according to manufacturer’s instructions. And then the tissue sections were incubated with primary antibodies [rabbit anti-USP36 antibody (14783-1-AP, Proteintech, 1:200) and rabbit anti-DOCK4 antibody (21861-1-AP, Proteintech, 1:100)] overnight at 4°C. Finally, DAB staining, hematoxylin redyeing and hydrochloric acid alcohol differentiation. The images were captured with a Nikon microscope imaging system (Nikon Ti-S, Tokyo, Japan) and analyzed by Image J software.

### Immunofluorescence Staining

The TECs were seeded on the chamber slides for 72 h. Cells were washed with PBS for three times and then fixed in 4% paraformaldehyde for 20 min at room temperature. After washing three times with PBS, cells were permeabilized in 0.5% Triton X-100 in PBS for 20 min at room temperature and blocked for 1 h at 37°C by 1% goat serum (Solarbio, SL038). Cells were then incubated with rabbit anti-USP36 antibody (14783-1-AP, Proteintech,1:200) overnight at 4°C. After washing with PBS, they were incubated with secondary antibody (Fluor488 Goat Anti-Rabbit IgG H&L, A32731, invitrogen, 1:1,000) for 1 h at 37°C. Nuclei were counterstained with DAPI for 10 min at room temperature. The images were captured with a Nikon microscope imaging system (Nikon Ti-S, Tokyo, Japan) and analyzed by Image J software.

### Immunoprecipitation Assay

To examine ubiquitination of DOCK4, immunoprecipitation assay (IP) was carried out using a Crosslink IP kit (26147, Thermo Scientific Pierce) according to the manufacturer’s instructions. In short, after treated with proteasome inhibitor MG132 (#2194, CST,10 nM) for 6∼8 h, cells were lysed with IP Lysis Buffer. Lysates (1 mg) were incubated at 4°C overnight with 3 μg (rabbit anti-DOCK4 antibody or rabbit control IgG antibody) and 20 μl Protein A/G Agarose on a rotator. The eluent was analyzed by western blotting and analyzed by Image J software.

### *In situ* Proximity Ligation Assay

The TECs were fixed in 4% paraformaldehyde and permeabilized in 0.75% Triton X-100. The PLA assay was carried out according to manufacturer’s instructions (DUO92101, Sigma) with rabbit anti-USP36 antibody (14783-1-AP, Proteintech, 1:100), rabbit anti-ubiquitin antibody (10201-1-AP, Proteintech, 1:100) and mouse anti-DOCK4 antibody (sc-100718, santa, 1:50). The signal was visualized using an imageXpress confocal microscope (Molecular Devices) and analyzed by Image J software.

### Mass Spectrometry-Based Proteomics

Differentially expressed genes with statistical significance between the two groups were identified through Volcano Plot filtering. The experiment consists of three sets of biological replicates. A total of 49 proteins were identified with a 95% confidence in two group cells, which were cultured in medium containing either 5 mM (normal glucose, NG) or 25.5 mM (high glucose, HG) glucose for 72 h. Genes were chosen for further data analysis such as clustering, GO, KEGG pathway, and protein interaction.

### Statement

The brightness of all the images has been increased by 50% using photoshop software.

### Statistical Analysis

Each experiment was performed at least for three times. All data were analyzed using Prism GraphPad 5.0, with Student’s *t*-test (comparison between two groups) or one-way ANOVA (comparison among multiple groups). Error bars are represented as mean ± S.D and statistical significance was denoted as follows: ^∗^*p* < 0.05, ^∗∗^*p* < 0.01, ^∗∗∗^*p* < 0.001, and ^****^p < 0.0001.

## Results

### USP36 in Renal Tubules of Diabetic Kidney Disease Patients and Mouse Models Are Significantly Increased

**FIGURE 1:**
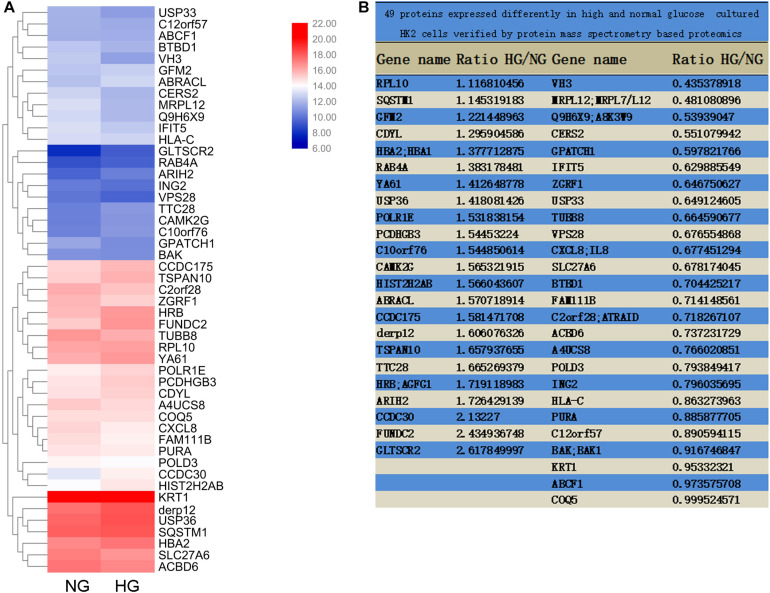
Mass Spectrometry-Based Proteomics. **(A)** The heatmap of M-S analysis. Compared with the 5 mM (normal glucose, NG) group, the expression of 49 proteins in 25.5 mM (high glucose, HG) group was significantly different. **(B)** The proportion of 49 protein expression in HG and NG.

**FIGURE 2:**
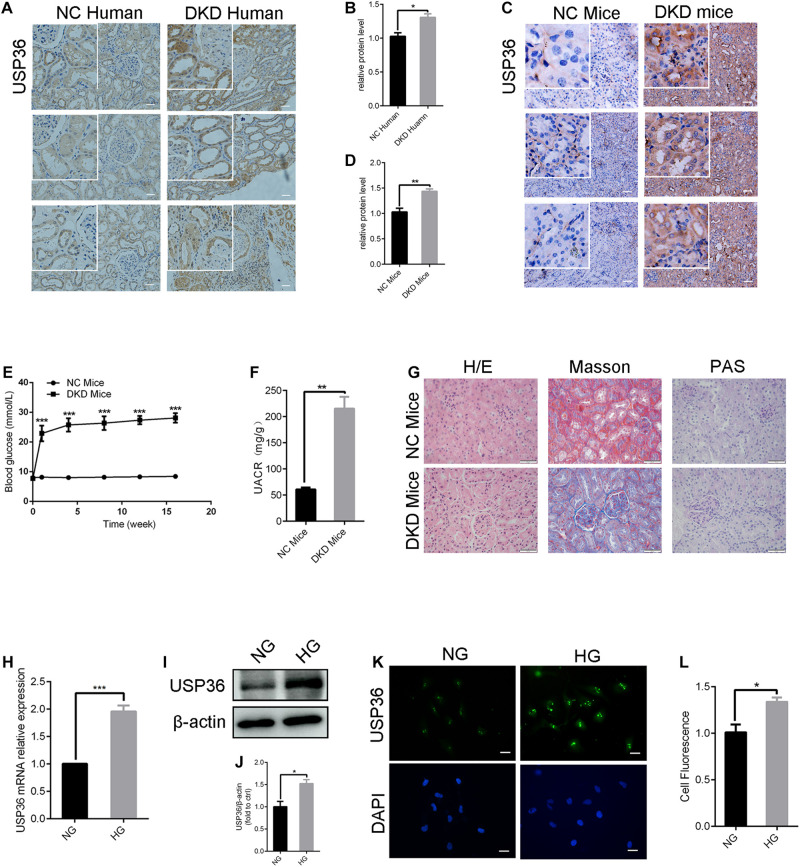
The expression of USP36 is significantly elevated under high glucose conditions in renal TECs both *in vivo* and *in vitro*. **(A,B)** Immunohistochemistry staining for USP36 expression in human kidney sections. NC (normal control) represented the renal histological sections from adjacent normal tissues of patients with renal cell carcinoma. Scale bars = 100 μm, *n* = 3. **(C,D)** Immunohistochemistry staining for USP36 expression in mouse renal tissues. Scale bars = 100 μm. NC (normal control) represented the renal histological sections from the normal male C57BL/6 mice, *n* = 3. Scale bars, 100 μm. **(E)** The blood glucose level in NC mice and DKD mice, *n* = 3, ***p < 0.001. **(F)** Urine albumin to creatine ratio (UACR) in STZ-induced DKD mice, *n* = 3, **p < 0.01. **(G)** Hematoxylin and eosin (H/E) staining, Masson’s trichrome (Masson) staining and Periodic acid-Schiff (PAS) staining in renals from STZ-induced DKD mice. Scale bars = 50 μm, *n* = 3. **(H,I,K)** qRT-PCR, western blotting and immunofluorescent staining were performed to detect the USP36 expression in high glucose induced TECs. Scale bars = 50 μm. Unpaired, two-tailed *t* tests were used, *n* = 3, ***p < 0.001. **(J,L)** WB and fluorescence analysis of the protein levels of USP36 in high glucose induced TECs. Data are presented as the mean ± S.D, *n* = 3, *p < 0.05.

To explore the pathogenic mechanism of diabetic kidney disease, we performed a mass spectrometry-based proteomics approach to search molecules that were differentially expression by high glucose in TECs. A total of 49 proteins were differentially expressed interval in high glucose-induced TECs and normal glucose controls TECs (Figures 1A,B), in which USP36 is significantly upregulated. Therefore, we were speculated that USP36 is likely to play a vital role in DKD. Immunohistochemistry staining was performed to detect the protein level of USP36 in renal tissues. Renal tissues from normal and DKD patients were used in our study. As shown in Figures 2A,B, the protein level of USP36 underwent significant elevated in human kidneys with DKD. To further explore the role of USP36 in DKD, we made a DKD mouse model by STZ treatment. The USP36 expression was also elevated in kidney tissues of STZ-induced DKD mice (Figures 2C,D). One week after STZ injection, the random blood glucose levels of mice were over 16.7 mM (Figure 2E). The kidney metabolic parameters of STZ-induced DKD mice was demonstrated by rose urine albumin to creatinine ratio (UACR) (Figure 2F). Compared with the control mice, the renal tubules vacuolation and mesangial matrix deposition were exacerbated in STZ-induced DKD mice through Hematoxylin and eosin staining, Masson’s trichrome staining and Periodic acid-Schiff staining examination (Figure 2G). These results indicated that USP36 was upregulated in DKD, which suggested that USP36 may be involved in the pathogenesis and development of DKD.

### USP36 in TECs Cultured in Diabetic-Like Condition Are Significantly Increased

As shown in Figures 2H–L, RT-qPCR, western blotting and immune-fluorescence staining analysis revealed a significant increase of USP36 in TECs treated with high glucose, which indicated the possible involvement of USP36 in the progression of DKD.

### USP36 Participates in the Regulation of Hyperglycemia-Induced EMT of TECs

**FIGURE 3:**
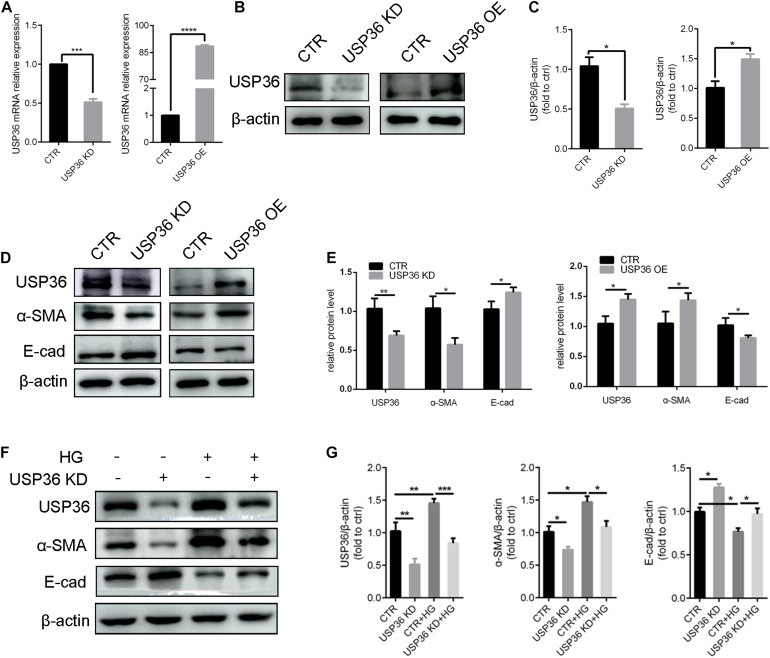
USP36 participates in the regulation of hyperglycemia-induced EMT in TECs. **(A)** RT-qPCR showed knockdown or overexpression efficiency of USP36 in TECs. Unpaired, two-tailed *t* tests were used, *n* = 3, ***p < 0.001, ****p < 0.0001. **(B,C)** WB analysis of the protein levels of USP36 after transfected with USP36-silencing siRNA or USP36-overexpressing plasmids. Data are presented as the mean ± S.D, *n* = 3, *p < 0.05. **(D,E)** WB analysis of the protein levels of USP36, α-SMA and E-cad after knockdown or overexpression of USP36. Data are presented as the mean ± S.D. *n* = 3, *p < 0.05, **p < 0.01. **(F,G)** WB analysis of the protein levels of USP36, α-SMA and E-cad in TECs exposed to long time high glucose following the knockdown of USP36. Data are presented as the mean ± S.D, *n* = 3, *p < 0.05, **p < 0.01, ***p < 0.001.

Epithelial-to-mesenchymal transition plays a considerable role in tubulointerstitial fibrosis, which is a characteristic of diabetic kidney disease (Zhao et al., 2017). In this study, whether USP36 has an effect on EMT under high glucose condition also attracted our attention. First, the cellular USP36 contents were either reduced or elevated by transfecting USP36-silencing siRNAs or overexpressing plasmid, and the transfection efficiencies were verified by RT-qPCR and western blotting (Figures 3A–C). Then, the epithelial marker E-cadherin and mesenchymal marker α-SMA were evaluated by western blotting after gain- or loss-of-function of USP36. As shown in Figures 3D,E, E-cadherin was significantly enhanced and α-SMA was obviously decreased by knockdown of USP36 in TECs. By contrast, USP36 overexpressing exhibited opposite effect. Finally, E-cadherin and α-SMA were evaluated by western blotting in TECs under high glucose. As shown in Figures 3F,G, E-cadherin was obviously decreased and α-SMA was significantly enhanced in TECs under high glucose, while knockdown of USP36 could effectively reverse the expression of E-cadherin and α-SMA, which suggesting that up-regulation of USP36 may be an upstream mechanism for EMT and diabetic tubulointerstitial fibrosis.

### USP36 Regulates the Ubiquitination Level of DOCK4

**FIGURE 4:**
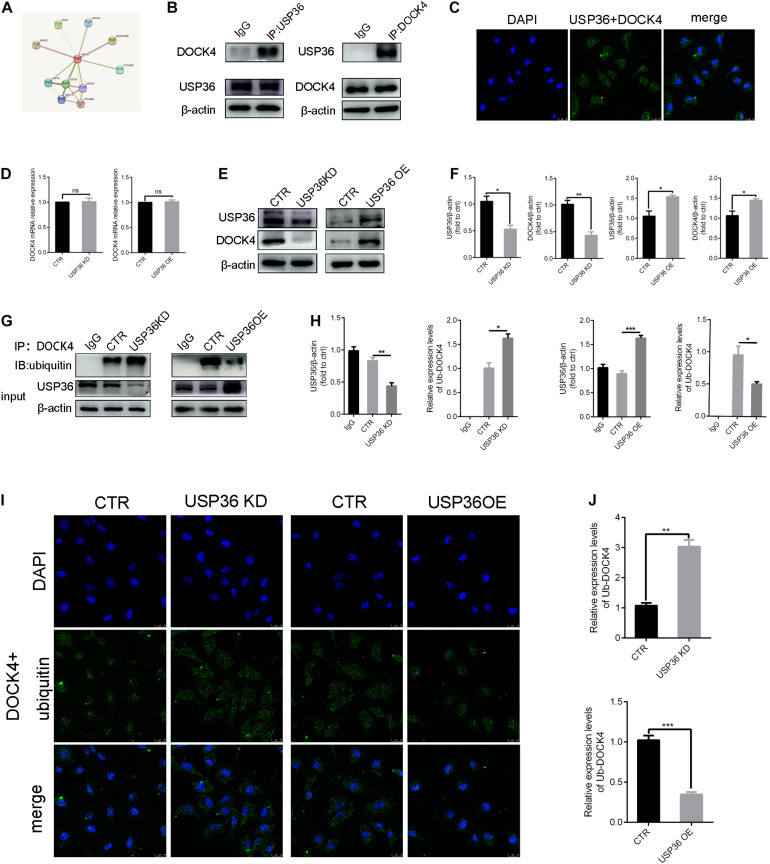
USP36 regulates the ubiquitination level of DOCK4. **(A)** String website was performed to predict that USP36 can bind to DOCK4. **(B)** Immunoprecipitation analysis was performed with cell lysates of TECs with anti-USP36 or anti-DOCK4 antibodies, *n* = 3. **(C)** Interaction of USP36 and DOCK4 in TECs visualized by Duolink proximity ligation assay, *n* = 3. **(D)** RT-qPCR analysis of the mRNA level of DOCK4 after knockdown or overexpression of USP36. Unpaired, two-tailed *t* tests were used to compare groups, *n* = 3, ns, *p* > 0.05. **(E,F)** WB analysis of the protein levels of USP36 and DOCK4 in TECs after knockdown or overexpression of USP36. Data are presented as the mean ± S.D, *n* = 3, *p < 0.05, **p < 0.01. **(G,H)** WB analysis of the protein levels of USP36 and the ub-DOCK4 in TECs after knockdown or overexpression of USP36. Data are presented as the mean ± S.D, *n* = 3, *p < 0.05, **p < 0.01, ***p < 0.001. **(I)** Ub-DOCK4 was detected by Duolink proximity ligation assay after knockdown or overexpression of USP36. Scale bars = 25 μm, *n* = 3. **(J)** Duolink proximity ligation assay analysis of ub-DOCK4 expression after knockdown or overexpression of USP36. Data are presented as the mean ± S.D, *n* = 3, **p < 0.01, ***p < 0.001.

As the regulation of EMT by USP36 was demonstrated, we continued to explore the underlying mechanisms. With this purpose, potential interactions with USP36 were speculated in string^1^ (Figure 4A). Among these proteins, particularly interest is DOCK4, due to its function in activating Wnt/β-catenin signaling pathway, which is associated with renal fibrosis (Xie et al., 2020). First, we evidenced that USP36 physically interacts with DOCK4 by IP and *in situ* proximity ligation assay (*in situ* PLA) (Figures 4B,C). To study the effect of USP36 on the expression of DOCK4, we evidenced the protein level of DOCK4 was significantly reduced or elevated by knockdown or overexpression of USP36 in TECs (Figures 4E,F). While the mRNA level of DOCK4 displayed no significant change (Figure 4D). Thus, we speculated that regulation of USP36 on DOCK4 expression through a post-translational mechanism. Ubiquitination is important for physiological processes which are controlled by ubiquitinating and deubiquitinating enzymes. Then, the data showed that the ubiquitination level of DOCK4 was significantly increased or decreased by knockdown or overexpression of USP36 in TECs (Figures 4G–J). Taken together, these results indicated that USP36 could regulate the ubiquitination level of DOCK4.

### USP36 Regulates EMT of TECs *via* DOCK4-Dependent Wnt/β-Catenin Signaling Pathway

**FIGURE 5:**
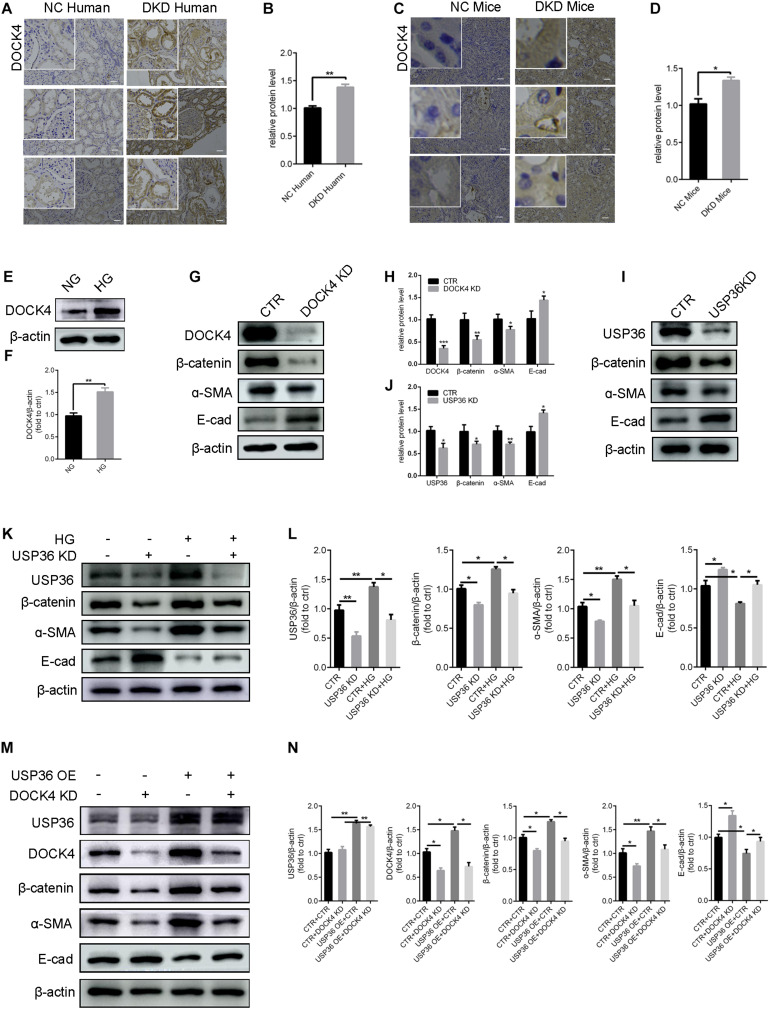
USP36 regulates EMT induced by high glucose of TECs *via* DOCK4-dependent Wnt/β-catenin signaling pathway. **(A,B)** Immunohistochemistry staining for DOCK4 expression in human kidney sections. Scale bars = 100 μm, *n* = 3. **(C,D)** Immunohistochemistry staining for USP36 expression in mouse renal tissues. Scale bars = 100 μm, *n* = 3. **(E,F)** WB analysis of the protein levels of DOCK4 in high glucose induced TECs. Data are presented as the mean ± S.D, *n* = 3, *p < 0.05. **(G,H)** WB analysis of the protein levels of DOCK4, β-catenin, α-SMA and E-cad in TECs after knockdown of DOCK4. Data are presented as the mean ± S.D, *n* = 3. *p < 0.05, **p < 0.01, and ***p < 0.001. **(I,J)** WB analysis of the protein levels of USP36, β-catenin, α-SMA and E-cad in TECs after knockdown of USP36. Data are presented as the mean ± S.D, *n* = 3, *p < 0.05, and **p < 0.01. **(K,L)** WB analysis of the protein levels of USP36, β-catenin, α-SMA and E-cad in TECs exposed to long time high glucose following the knockdown of USP36. Data are presented as the mean ± S.D, *n* = 3, *p < 0.05, and **p < 0.01. **(M,N)** WB analysis of the protein levels of USP36, DOCK4, β-catenin, α-SMA, and E-cad after co-transfected with USP36-overexpressing and DOCK4-silencing siRNA. Data are presented as the mean ± S.D, *n* = 3, *p < 0.05, and **p < 0.01.

Previous studies demonstrated that DOCK4 is responsible for the degradation of β-catenin (Xie et al., 2020). Under this study, whether USP36 has an effect on EMT through DOCK4-dependent Wnt/β-catenin signaling pathway in TECs under high glucose condition has aroused our attention. Consistent with USP36, immunohistochemistry staining and western blotting analysis also revealed a significant increase of DOCK4 both *in vitro* and *in vivo* under high glucose condition (Figures 5A–F). The expression of β-catenin and α- SMA were significantly reduced and E-cad was obviously increased when knockdown of DOCK4 or USP36 in TECs (Figures 5G–J). As shown in Figures 5K,L, western blotting analysis revealed that the expression of β-catenin and α- SMA were significantly increased and E-cad was obviously reduced in high glucose-induced TECs, while these protein expressions were partial reversed by USP36 knockdown. The results revealed that DOCK4 knockdown effectively abolished USP36 overexpression-induced EMT through suppressing Wnt/β-catenin signaling pathway in TECs (Figures 5M,N). In brief, USP36-mediated deubiquitination of DOCK4 could contribute to the EMT in diabetic kidney disease *via* the Wnt/β-catenin signaling pathway.

## Discussion

Ubiquitination and deubiquitination play a key role in maintaining cell homeostasis and are involved in the regulation of a number of biological processes. Our previous study demonstrated that as a deubiquitinating enzyme, USP36 protects TECs cells from ischemic injury by stabilizing c-Myc and SOD2 (Liu et al., 2019), implying a potential role of USP36 in renal pathology. In this study, we revealed the increased expression of USP36 in high glucose-induced renal tubular cell injury both *in vitro* and *in vivo*, and the role of USP36 overexpression in DKD is related to its deubiquitination effect of DOCK4, that could activate Wnt/β-catenin signaling pathway and mediate EMT. Thus, these results provide evidences for a novel role of USP36 in the pathogenesis of diabetic kidney disease and identify a new target protein that is deubiquitinated by USP36.

To date, little studies concerning the ubiquitin system in the pathogenesis of DKD has been reported, moreover, these limited studies reached controversial conclusions. On the one hand, the ubiquitination activity seems to be enhanced in DKD. For example, the ubiquitin fusion protein UbA52, mainly localized in kidney tubules, was found to increase in patients with diabetes (Xie et al., 2020). Further, the expression of UBE2v1, a ubiquitin-conjugating E2 enzyme variant 1, was also increased in DKD patients and in renal tubular cells under high glucose condition, along with α-SMA up-regulation (Pontrelli et al., 2017). In diabetic kidney, the activity of E3 ligase c-Cbl was increased and thus resulted in podocyte injury by promoting nephrin degradation and reducing CD2AP expression (Teng et al., 2016). USP22, a deubiquitinase of USP family, was decreased in glomerular mesangial cells treated with advanced glycation end products (AGEs) (Huang et al., 2015). However, on the other hand, the enhanced process of deubiquitination has also been reported to participate in the diabetic pathology. UCH-L1, a member of the UCH protease family that deubiquitinates ubiquitin-protein conjugates, was raised in the kidneys of diabetic patients and seemed to be related to the development of DKD (Zhang et al., 2016). In STZ-induced DKD animal model, administration of proteasomal inhibitor MG132 had renal protective effect *via* inducing Nrf-2 levels (Cui et al., 2013). Taken together, results of these studies indicate that the ubiquitination and deubiquitination are extremely complex processes in diabetes, which seems to be cell type-specific and protein-specific. Thus, we employed proteomic analysis to explore the key proteins involved in the high glucose induced renal tubular cell changes in ubiquitin system, and USP36 emerged as a candidate considering its 1.4-fold increase.

A line of proteins had been demonstrated to be targets of USP36-induced deubiquitination, including c-Myc (Sun et al., 2015b), superoxide dismutase 2(SOD2) (Kim et al., 2011), H2B (DeVine et al., 2018), PME-1 (Kim et al., 2018), DHX33 (Fraile et al., 2018), NPM, and FBL (Endo et al., 2009a). In this study, additional potential interactions of USP36 were speculated (Figure 4A). Particular interest is DOCK4, a guanine nucleotide exchange factor (GEF), on account of its essential role in Wnt/β-catenin signaling (Upadhyay et al., 2008) which is associated with EMT (Xie et al., 2020). However, the way that USP36 regulates a protein expression is not limited to its direct interaction and deubiquitination modification. It has been reported that USP36 is critical for ribosomal RNA synthesis and mRNA translation through the regulation of RNA polymerase I stability (Endo et al., 2009b). In addition, USP36 has been identified as a deubiquitinase for H2Bub1, which is a monoubiquitination of histone H2B enriched at actively transcribed genes and positively correlated with their expression levels (DeVine et al., 2018). Nevertheless, in this study, we demonstrated that USP36 regulates DOCK4 expression most possibly through its deubiquitinating enzyme activity, instead of an direct regulation of transcription or translation. To elucidate the interaction between DOCK4 and USP36, immunoprecipitation and Duolink proximity ligation assay were employed (Figures 4B,C). Results showed that the DOCK4 and USP36 are more likely to have a direct connection, and ubiquitinated form of DOCK4 changed just in opposite to USP36, indicating that DOCK4 is a target of USP36 (Figures 4G,I). Furthermore, we have demonstrated for the first time that DOCK4 has a ubiquitinated form and regulated by USP36. Yet, more comprehensive and in-depth researches are needed.

DOCK4 is a member of DOCK family proteins, which are conserved across different mammalian species and emerged as a novel class of Rac/Cdc42 GTPase guanine nucleotide exchange factors (GEFs). However, members of DOCK family (termed DOCK1 to DOCK11) have been implicated to play distinct roles in diverse cell type-specific processes (Gadea and Blangy, 2014). As for DOCK4, it is a 225 KDa protein with multiple signaling/protein-protein interaction domains (Upadhyay et al., 2008). Mutations or reduced expression of DOCK4 is related to malignancies in breast (Kobayashi et al., 2014), lung (Yu et al., 2015), brain (Debruyne et al., 2018) and blood tissues (Sundaravel et al., 2019) as well as tumor metastasis (Hiramoto et al., 2006). Dysfunctional DOCK4 is also involved in several neuropsychiatric disorders, including autism, dyslexia, mild intellectual disability (Huang M. et al., 2019), schizophrenia (Akahoshi and Yamamoto, 2018) and hearing impairment (Uehara et al., 2015). In addition, DOCK4 boosts atherosclerosis *via* internalization of SR-B1 and transport of LDL (Huang L. et al., 2019). However, very little is known with respect to the impact of DOCK4 in diabetes. Our present study revealed the increased DOCK4 expression both *in vivo* and *in vitro* in circumstance of high-glucose, while knockdown of DOCK4 could reverse the overexpression of USP36 induced EMT effect, indicating a role of DOCK4 played in diabetic renal fibrosis. EMT plays a considerable role in tubulointerstitial fibrosis, which is a characteristic of diabetic nephropathy. The process of EMT contains loss the expression of E-cadherin and increased the expression of α-smooth muscle actin (α-SMA) (Bedi et al., 2008). Under high glucose condition, previous study reported that the expression of β-catenin was increased and have the ability to promote EMT (Guo et al., 2014). Since DOCK4 has been demonstrated to act as a scaffold protein and impedes β-catenin degradation through enhancing the release of β-catenin from degradation complex (Upadhyay et al., 2008), the regulation of EMT by DOCK4 is possibly a result of its role in modifying Wnt/β-catenin signaling. Taken together, our have found that USP36-mediated deubiquitination of DOCK4 contributes to the diabetic kidney disease *via* the Wnt/β-catenin signaling pathway.

In summary, our results have allowed us to identify USP36 as an essential gene in diabetic renal tubular injury through, at least in part, DOCK4-dependent Wnt/β-catenin signaling pathway. We describe for the first time the regulatory role of USP36 on the ubiquitination levels of DOCK4, which constitutes a new mechanism of USP36 functional implication in diabetic pathology.

## Data Availability Statement

The original contributions presented in the study are publicly available. This data can be found here http://proteomecentral.proteomexchange.org/cgi/GetDataset?ID=PXD023347, accession number: PXD023347.

## Ethics Statement

The studies involving human participants were reviewed and approved by the investigations were conducted in accordance with the principles of the Declaration of Helsinki and were approved by the Research Ethics Committee of Shandong University after informed consent was obtained from the patients. The patients/participants provided their written informed consent to participate in this study. The animal study was reviewed and approved by all experiments were approved by Institutional Animal Care and Use Committee of Shandong University (No. ECSBMSSDU 2018-1-045). Written informed consent was obtained from the individual(s) for the publication of any potentially identifiable images or data included in this article.

## Author Contributions

SZ contributed to the design of the project, performed most of the experiments, data analysis, and prepared the manuscript. SH performed the immunohistochemistry experiments. YL performed real-time polymerase chain reaction experiments. WS and TD performed proteomics data analysis. ZC contributed to the design of the project. HF and QW helped modify the manuscript. All authors read and approved the final manuscript.

## Conflict of Interest

The authors declare that the research was conducted in the absence of any commercial or financial relationships that could be construed as a potential conflict of interest.

## Footnotes

1 www.string-db.org
